# Preventing Plague, Bringing Balance: Wildlife Protection as Public Health in the Interwar Union of South Africa

**DOI:** 10.1353/bhm.2021.0054

**Published:** 2021

**Authors:** Jules Skotnes-Brown

**Keywords:** environmental health, animal history, history of epidemiology, bubonic plague, economic zoology, southern African history, environmentalism, wildlife protection

## Abstract

This article proposes a new line of enquiry in the history of animal conservation by suggesting that African wildlife protection was a form of public health in the early twentieth century. Through examining the activities of South African epidemiologists, politicians, bureaucrats, farmers, and zoologists in the 1920s and 1930s, the author argues that wildlife was integrated into epidemiological strategies and agricultural modes of production. Against the backdrop of a series of plague outbreaks, carnivora once deemed “vermin” were legally protected as sources of human health and agricultural wealth. As public health, food security, and carnivore populations were imbricated, the categorical boundaries between human and animal health also began to blur. Ultimately, this case suggests the need to bridge environmental and medical history and to broaden the history of environment and health beyond canonical figures such as Rachel Carson. Paying attention to colonial “peripheries” and African thought is critical in understanding the origins of twentieth-century environmentalism.

[Other P-464]

Without birds, “the most beautiful and loveable of God’s creation,”^1^ wrote Frederick FitzSimons, “It is questionable whether” humankind “could even manage to exist, except in a miserable way.”^2^ FitzSimons, a South African public intellectual, museum director, and economic zoologist, was referring not to the brilliant colors birdlife brought to the veld, nor to the charm of their melodic voices, but to their indispensability to public health. In his opinion, without its abundance of birds, the earth would be reduced to a barren desert, and those destitute human survivors would be at the mercy of malarial mosquitos, blood-sucking flies, and plague-carrying rodents. In “waging incessant war on the insects which carry disease microbes to man and beast,” birds rendered humankind “a service which cannot be overestimated.”^3^

Roughly one hundred years ago, the bird protection movement spread across the world in response to a perceived global decline in avian fauna. The chief culprits of this crisis were often thought to be farmers, who destroyed wilderness for large agricultural estates, and defended these from bird depredations with poisoned grain and shotgun shells. Today, the earth faces two strikingly similar crises: the global collapse of insect populations and the spillover of zoonotic diseases from animal to human populations as forests and jungles are cleared for agriculture.^4^ Most recently, COVID-19 has brought the latter into unprecedented light. The degradation of nature in the relentless pursuit of economic growth, we are constantly reminded in articles and op-eds, has become a serious risk to human health. This is particularly the case in Africa, where past outbreaks of Ebola and fears of future undiscovered zoonotic diseases haunt the pens of conservationists and epidemiologists alike.^5^ In this current ecological [Other P-465] and medical crisis, it is urgent that we historians turn our attention toward the relationship between wildlife, agriculture, and human health.

Most histories of environment, wildlife, and health in colonial circumstances have demonstrated how Europeans *altered* environments in order to render them “healthy” through sanitation, vector eradication, and public health policies.^6^ The counterpart to this historiography, which addresses environmental *preservation* and health, has received less attention, and has typically been associated with the thinking of North Americans such as Aldo Leopold.^7^ Biologists in the interwar period, as Gregg Mitman has argued, sometimes looked to Hippocrates and his ideas about health being “a state of equilibrium between the organism and its total environment.”^8^ Proponents of this thought worried that environmental alteration would upset equilibrium, and produce ill health in humans, animals, and the land itself. These ideas became particularly important in the post–World War II West, as environmentalism gained momentum in the wake of Rachel Carson’s *Silent Spring*.^9^ In wildlife management, Harriet Ritvo has argued that by the second half of the twentieth century, the “standard unit of management” become the “ecosystem rather than the species.” This led to the protection of carnivora, who were previously considered to be “blots on the landscape.”^10^ Yet wildlife management by ecosystem was not the first time wildlife managers broadened the scope of conservation beyond the species: from the 1910s in South Africa, the idea of a “balance of nature” was, in many cases, a key unit of management. Although this concept was considerably different to the ecosystem, its devotees nevertheless posited that each creature played a role in maintaining, or disrupting, nature’s equilibrium, and decisions about wildlife management needed to be made in a holistic framework. [Other P-466]

In 1910s–30s South Africa, concern about environmental alteration threatening human health was growing and influenced public health strategies. Such concerns have received little attention in southern African environmental and medical history, which has focused primarily on the separation of humans and wildlife. This, the argument goes, was achieved by creating game reserves and exterminating animals on rural frontiers.^11^ While this was sometimes the case, not all farmers and biologists were convinced that farms should or *could* be separated from “natural” environments. The possibility that living wild animals might have been integrated into public health strategies as a source of human health remains largely unexplored, due to the presence of a well-studied state veterinary department that, according to Jane Carruthers, Shirley Brooks, and Karen Brown, was antagonistic to wildlife protection. Medical and veterinary scientists, these scholars argue, were opposed to wildlife protection on the grounds that wild animals were reservoirs of disease. For them, wildlife destruction was a means of maintaining livestock health.^12^

Through examining the attempts of employees and affiliates of the Union of South Africa’s Department of Public Health to control bubonic-plague-carrying rodents in the veld, this paper examines an unstudied approach to wildlife management that rested on the opposite assumption. For the first Secretary of Public Health, James Alexander Mitchell, and a series of zoologists and “rodent inspectors” with whom he worked, wild animals were not simply reservoirs of disease. Rather, numerous carnivora provided public health services to humankind in consuming plague-carrying rodents, and insect carriers of other diseases. The protection of such wildlife was fundamentally a matter of human health. Such ideas were not Mitchell’s own but were derived primarily from the propaganda publicized [Other P-467] by Frederick FitzSimons, director of the Port Elizabeth Museum. Between 1920 and 1923, FitzSimons pieced together a composite of U.S. and British research into economic ornithology—a discipline that attempted to calculate the utility, harmfulness, or neutrality of birds to agriculture—along with indigenous African wildlife management technologies, and crudely applied these to a *white* South African milieu. In the 1920s and 1930s, in attempt to control “veld rodent” vectors of plague in the countryside, Mitchell, in collaboration with FitzSimons and zoologist Austin Roberts, attempted to agitate the Union government to criminalize the killing of “useful” rodent-devouring birds of prey, wildcats, and mongooses and to teach farmers on the ground that these carnivora were not their “enemies” but their “friends.” Although their views were not entirely aligned, FitzSimons, Roberts, and Mitchell suggested that in a country transformed by mechanized agriculture and urbanization, birds of prey and terrestrial carnivora played a critical role in maintaining the “balance of nature” and preventing environmental and medical catastrophes.

Such ideas were divisive and prompted a series of debates in parliament, periodicals, and correspondence over the degree to which birds of prey and terrestrial carnivora brought balance to nature and prevented outbreaks of plague. How nature’s balance should be maintained, the role humans should play in keeping animal populations in balance, and which wild animals could be considered agents of public health provoked a wide array of responses from individuals of diverse linguistic, professional, and racial backgrounds. Although publicists of wildlife protection as a measure of public health appear to have been primarily white, English-speaking, and urban, their publications and lectures drew supporters and critics from rural Anglophones, Afrikaners, and Africans alike. Despite such patchy reception, wildlife protection as a public health strategy nevertheless shaped the South African environmentalist movement. By the 1930s, in numerous parts of the country, all owls, several hawks and eagles, as well as wildcats, once considered “vermin,” became legally protected sources of public health. As human and predator population health and wealth were imbricated, so too did the categorical boundaries between human and animal roles in the economies of nature and agriculture begin to blur. While predators of numerous species were retheorized as farm laborers and public health workers, Roberts and Mitchell attempted to transform farmers into rodent predators in an extensive rodent-destruction campaign.

Ultimately, this paper suggests a need to bring the history of natural history, medical history, and environmental history into conversation when thinking about the origins of twentieth-century environmentalism. As Sellers [Other P-468] has argued, until relatively recently, the “main currents in medical and environmental history” have run “in opposing directions.”^13^ This paper seeks to bridge these by suggesting a new avenue of historical enquiry in African history: wildlife protection not only as an ethical imperative, a form of national heritage, a means to preserve “the hunt,” or a strategy to create biological laboratories, but also as a form of public health.^14^

## Bubonic Plague as a Symptom of Nature Out of Balance

In early 1920, James Alexander Mitchell, the first secretary of the Department of Public Health (formed in 1919), was baffled by what he later described as a “complete mystery.”^15^ Since 1914, outbreaks of bubonic plague, one of the most dreaded diseases in history, had been appearing in isolated, disconnected, and seemingly random locations across the Orange Free State, Cape, and Transvaal provinces. Confoundingly, these were areas in which “ordinary domestic rodents” that had ravaged Cape Town, Port Elizabeth, and Durban in 1900–1901 were few or absent, and no evidence of infection in wild veld rodents—who Mitchell suspected might be harboring the disease—was visible either.^16^

The reappearance of bubonic plague far into the interior of the country formed the latest in a series of medical and environmental ailments with which the newly created Department of Public Health had to contend. Mitchell had taken up his post in a period when South Africa was reeling from the ecological consequences of a series of profound political changes in the late nineteenth and early twentieth centuries, which shaped his understanding of public health. In 1902, the Boer War ended with the British annexation of two independent Boer republics.^17^ According to Mitchell, the conflict was responsible for the importation of plague into the country: it had greatly increased the demand for “Hay, forage, [Other P-469] and grain” from “plague-infected ports such as Buenos Ayres, Rosario, Rio de Janeiro, Santos, Bombay, Madras, Karachi and Mauritius.”^18^ In 1910, the four colonies (Cape, Natal, Orange Free State, and Transvaal) unified, forming the Union of South Africa. This was an uneasy union, characterized by divisions and resentment, and further complicated by the patchwork nature of the state: the former colonies became provinces and retained considerable controls over legislation, including wildlife protection laws.^19^ The unification of the “white races” accelerated the exclusion of African people. In 1913, the Natives Land Act was passed, allocating just 7 percent of the land to the African-majority population.^20^ Much of this land was antithetical to agriculture and settlement, leading African National Congress founding president John Dube to protest that African reservations in Zululand were “full of” with malarial “fever and nagana.”^21^

Accompanying such political changes came environmental changes. Since the mid-nineteenth century, state officials in the Cape of Good Hope had attempted to implement “progressive” or “rational” agricultural techniques to optimize food production.^22^ By the end of the century, this “model of scientific agronomy spread northwards” toward the Transvaal and Orange Free State governments.^23^ Advocates of “progressive” agriculture—self-styled “progressives”—were primarily English, but also included some Afrikaners and colonial-educated Africans.^24^ “Progressive” farmers mobilized the sciences to optimize food production and combat livestock diseases, soil erosion, and pests.^25^ They scorned or ignored many existing farming methods practiced by Africans and settlers alike and in particular the long-established migratory farming practice that William Beinart refers to as transhumance.^26^ Transhumant farmers had [Other P-470] once avoided malarial or infertile areas, moved cattle seasonally, and controlled pests as they desired.^27^ “Progressives” dismissed transhumance as environmentally damaging and a source of the spread of disease.^28^ Instead of migrating to avoid environmental problems, and kraaling animals at night, they insisted that these should be tackled in situ by creating large enclosed farms supported by mechanization and irrigation schemes, fodder crops, monocultures, and a declaration of war on insects, rodents, parasites, and vermin.^29^

Progressives had a “disproportionate influence over state policy in certain spheres”^30^ and their project of “modernizing” agriculture accelerated in the late nineteenth to mid-twentieth centuries. The enclosure of farms was legislated in the Cape in 1883, and with the rapid expansion of livestock farming between 1905 and 1930, agriculture became an increasingly important source of state revenue.^31^ In the 1910s and 1920s, “inefficient” farming practices were blamed for drought and soil erosion and deemed in need of modernization.^32^ State interventions were imposed in African reserves, which were “insensitive to rural social relationships and often highly disruptive.”^33^ The enclosure and mechanization of agriculture across much of the countryside likewise created a class of “poor whites,” who lacked the capital to compete with mechanized farms.^34^ In the 1920s and 1930s, these “poor whites” were a key concern in both Union politics and eugenic societies. Whites living on rural frontiers, many worried, were racially degenerating on account of their exposure to a taxing climate, [Other P-471] insect-borne diseases like malaria and bilharzia, and proximity to putatively “primitive” people.^35^

The process of “modernizing” agriculture brought new vigor to a long-standing conflict between settlers and “vermin.” Between the 1890s and 1930s, under the direction of veterinarians and zoologists, quarantine zones between farms and the wilderness were created across the colonies/provinces in which vegetation and wildlife was eliminated, and “vermin” found trespassing on enclosed farms was continuously killed.^36^ The increasing visibility of pests produced anxieties for the new state. Swarms of locusts appeared to intensify, tearing down vegetation as they feasted.^37^ Disease was rampant and often a direct result of industrialization or large-scale agriculture. In 1918, the influenza pandemic decimated the country, reducing the mining workforce, the backbone of the economy, to 62 percent capacity.^38^ In the 1910s and 1920s, nagana, a livestock disease that caused progressive emaciation and death in cattle, appeared to be spreading with intensity.^39^ Malaria remained a serious problem in the subtropical Natal province and parts of the Transvaal.^40^ But of all of these maladies facing Mitchell and the Department of Public Health, bubonic [Other P-472] plague, a disease that was “rendered an object of knowledge under the bane of its perceived ability to wipe out humanity,”^41^ was perhaps the most terrifying and required urgent attention.^42^ Two outbreaks of plague on farms called Grootdraai (in February 1920) and Angra Pequina (in March 1920) near Bothaville in the Orange Free State provided the first opportunity for the Public Health Department to investigate. Austin Roberts, a zoologist affiliated with the Transvaal Museum, was commissioned to perform a seemingly simple job: find and destroy the rodents responsible.^43^ Roberts was a higher vertebrate zoologist who is today considered to be the most influential South African ornithologist and whose field guide to birds is still in print.

Roberts arrived in Bothaville on April 30, 1920, and swiftly commenced his studies at Angra Pequina. During his nearly three months of fieldwork on the farm, he battled to complete his task. Excavating the soil in search of burrows was exhausting work, and trapping was almost futile—carnivores devoured caught rodents while he slept. All he could offer was circumstantial evidence that rodents in the area such as the springhaas, gerbille, and multimammate mouse were reservoirs of the disease.^44^ Subsequent investigations by rodent catcher William Powell and a team of doctors and laborers confirmed his speculation: hundreds of infected gerbilles and multimammate mice were discovered on the farm.^45^ The results of such investigations were disturbing: thousands of rodents lay hidden under the soil, harboring a terrifying infection, and even for a zoologist with knowledge of their habits, these could be detected only by “almost microscopic examination of the ground.”^46^

Throughout the 1920s, Powell conducted a series of rodent surveys across the Union to determine the prevalence of plague, and areas vulnerable to the disease on account of their veld rodent populations. [Other P-473] By 1925, he had made alarming findings: plague and susceptible rodents were scattered not only in small pockets but over a vast area, spanning much of the countryside in the Orange Free State, Transvaal, and Cape provinces. More worryingly, these rodents appeared to be encroaching upon densely populated cities.^47^

Although Powell had identified and roughly charted the distribution of the reservoirs, the origins of the outbreaks and the factors driving the spread of plague remained obscure. Why had this disease reappeared approximately twenty years after its apparent eradication far into the interior of the country, and not at its coastal epicenters of Cape Town, Port Elizabeth (now Gqeberha), and Durban? And how could one ever hope to control a zoonotic disease harbored by minute, superabundant rodents and their fleas across hundreds of kilometers of barren veld? Even though Mitchell was a veteran of plague control who had been involved in containing the 1900–1901 outbreak in Cape Town,^48^ his experience was largely useless in the veld. Urban-plague-control policies in South Africa largely gravitated around attempts to exclude rats from areas of human occupation through retrofitting houses to render them rat proof, razing entire buildings to the ground and burning their contents, forcibly removing Africans and Asians from their homes on suspicion that these harbored rats or fleas, inspecting and fumigating rat-occupied premises, and strategically laying out poisons and traps.^49^ Such spatial strategies of urban rodent exclusion, however, could not be utilized in the vast expanse of the veld: although one could rat proof a farmer’s house, a field of maize was another matter. Perhaps fortuitously for Mitchell, zoologist Frederick FitzSimons, the ringleader of a group of aggressive bird protection publicists of South Africa, had already provided a strategy for excluding rodents from settler farms: the labor of wild birds. [Other P-474]

## Wild Birds as Agricultural Laborers and Public Health Workers

By the 1920s, FitzSimons was a household name in his local city of Port Elizabeth and among readers of the farming periodical *Farmer’s Weekly*. An Irish-born zoologist whose family settled in South Africa in 1877, FitzSimons is largely remembered for his antidote kit for South African snake venom and his endeavors to popularize natural history.^50^ His Port Elizabeth Museum was visited by approximately 100,000 to 150,000 people per year and was a lively center of natural history in the Cape Province.^51^ Seeking to attract audiences of white and “coloured”^52^ people from across the region, FitzSimons and his wife Henrietta FitzSimons regularly hosted evening lectures, invited schoolchildren to the museum, and conducted lecture tours across the Cape Province.^53^ FitzSimons was involved with municipal public health in Port Elizabeth and in the 1910s used his position to conduct a propaganda campaign against the housefly, which he saw as a vector of typhoid, influenza, and other infections.^54^ In the 1910s and 1920s, he also wrote a regular column on South African natural history in *Farmer’s Weekly*: an agricultural newspaper that was primarily directed at an English-speaking audience of “progressive farmers,” but nevertheless had both Afrikaans and African readers.^55^ These articles formed the nucleus of his four volumes on *The Natural History of South Africa:* [Other P-475] *Mammals* (1919–20) and two volumes on *The Natural History of South Africa: Birds* (1923). In these articles, and in his museum, he hoped to nurture a love of nature among farmers and propagate the need to protect species threatened with extinction.^56^

In his written and oratory work of the 1920s—articles published in daily newspapers and *Farmer’s Weekly*, a series of monographs, lectures delivered across the country, and exhibited material in the Port Elizabeth Museum—FitzSimons prophesized a looming environmental apocalypse. Decades of persecution of South African wild birds, under an erroneous belief that they were pests, or simply sheer bloodlust, he argued, had upset the “balance of nature” and wrought environmental and medical chaos. Once in healthy equilibrium, balance had been tipped, resulting in biblical plagues of locusts, rodents, flies, as well as livestock and human diseases.^57^ In direct opposition to many veterinarians, FitzSimons argued that wildlife protection was not antithetical to the agrarian economy; rather, wildlife destruction was. Insects and rodents, in his opinion, were the chief cause of all South Africa’s medical and agricultural woes and “enemies” of humankind. Armies of locusts, rats, mosquitos, and caterpillars were locked in perpetual warfare with “man” and sought to drive “him” from “the face of the earth.”^58^ With fewer predators and an increased supply of food in the form of vast maize and mealie monocultures, this panoply of pests were breeding prolifically, devouring crops, and broadcasting plague, trypanosomiasis, malaria, bilharzia, and other diseases far and wide. Fortunately for humankind, these “enemies” were kept in check by powerful allies of humankind: wild birds.^59^

Seeking to appeal directly to capitalist farmers, FitzSimons framed this argument in terms of goods and services. Drawing upon the writings of British gentleman James Buckland, he argued that birds should be, quite [Other P-476] literally, integrated into agricultural modes of production. In consuming insects, rodents, and weed seeds, he argued, these birds were providing a service for farmers. Usually, their services were provided gratis, but on occasion the bird would claim some of the farmers crops as payment for her efforts.^60^ According to FitzSimons, far from being thanked by farmers for services rendered in pest control and public health, wild birds were instead slaughtered in enormous numbers.

Such actions were dangerous not only to the farming community but to citizens of the entire country. Instead of trying to exterminate birds and sever themselves from nature, FitzSimons thought a much safer strategy was for farmers to ally themselves with wild birds by engaging in bird “preservation and protection.”^61^ To exclude insect and rodent pests from their lands, they would need to accommodate birds. To do this, they could integrate bird labor into farming strategies by planting forests, creating reservoirs, and providing nesting boxes. If farmers experienced bird depredations, they could use nonviolent methods to scare them, such as rattles, scarecrows, and laborers patrolling orchards with dogs.^62^ FitzSimons presented these methods as a scientific approach that applied natural history to agriculture and offered an alternative to vernacular pest control. However, many of these techniques had already been in use by indigenous African peoples. For example, in the Transvaal, the Balobedu utilized various nonviolent (or less violent) technologies for scaring crop-eating birds.^63^ One, according to anthropologist Eileen Krige, involved killing a hawk or an owl, and putting it on a tree to act as a scarecrow.^64^ Another involved “tying string to sticks planted all over the field to which [Other P-477] calabashes or tins containing stones are hung. When the string is pulled all the small stones rattle making sufficient noise to scare the birds away.”^65^ Both strategies were presented by FitzSimons as his own in 1922.^66^

**Figure 1:**
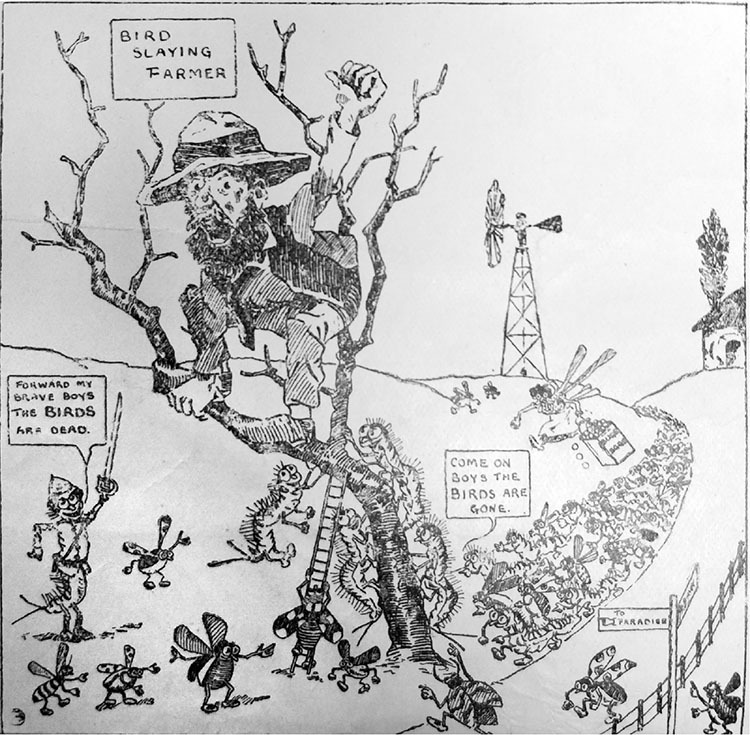
Cartoon by H. F. James depicting a stereotypical “poor white,” “bird-slaying” farmer in a pamphlet accompanying a FitzSimons lecture in Cape Town. From “Bird Life of South Africa, Mr. FitzSimons’ Plea for Preservation,” 1927, KAB, PAN 2/1.

Whether intentionally or not, FitzSimons was borrowing these ideas from the Balobedu, yet he condemned African farmers as bloodthirsty, backward, and a bad influence on rural whites. Tapping into omnipresent fears of white degeneration in the countryside,^67^ he argued that bird destruction was also hindering the racial “progress” of the country. Through exercising violent brain “lobes” shared with our simian ancestors, “bird slaying”^68^ farmers were allegedly “lower than an animal” and rapidly degenerating toward what he regarded as the lesser status of Africans.^69^ Playing into the ever-present discourse of “Black Peril” he dismissed Africans as cruel peoples who destroyed “immense numbers of birds,” to the benefit of vermin, and detriment of whites (see Figure 1).^70^

Ultimately at stake was not merely the extinction of birdlife, but the collapse of white society and the extinction of humankind in Africa. Thus, for FitzSimons, birds played a critical role in maintaining public health and agricultural wealth: bird protection was fundamentally a matter of human bodily, national, and racial health. With only a handful of exceptions,^71^ South African wild birds were boons granted by nature who maintained ecological stability. If insects and rodents too greatly outnumbered wild birds, equilibrium would be destroyed beyond nature’s ability to balance itself, and the earth would be reduced to an apocalyptic wasteland, populated only by those he considered to be subhuman. To prevent this calamity, humans had to stop obstructing nature’s balancing effect by offering protection to virtually all wild birds. Nature itself was thus fundamentally hospitable to white farmers, and to allow it to balance itself, all they had to do was cease shooting birds.

Such ideas were controversial, and although they were praised by some farmers and zoologists, they were hotly contested by others who insisted that farmers were the experts on their own farms, and knew which species were helpful or otherwise. FitzSimons was inundated with requests [Other P-478]

to lecture at schools and farmers associations.^72^ He was swamped with twelve to twenty letters *per day* from farmers interested in his methods.^73^ Numerous farmers pushed back against him, publishing their own rebuttals in *Farmer’s Weekly* between 1920 and 1926. Some insisted that many of [Other P-479] the birds FitzSimons wanted to protect were mostly pests that decimated their fields and attacked their livestock. Bird behavior, to such farmers, was adaptable, and ontologies of pestilence could not be standardized: some formerly helpful birds had become pests precisely because of the broad transformations in agriculture, and moreover, a grain farmer’s bird ally could be a chicken farmer’s enemy.^74^ Others speculated that the insects that FitzSimons so viciously attacked might also serve some broader purpose in maintaining balance.^75^ Farmers were not alone in their critiques. Zoologist Austin Roberts supported such perspectives and dismissed FitzSimons as a fanatical pseudoscientist who made recommendations without evidence and unjustly attacked farmers.^76^

On the other end of the scale, numerous farmers praised his methods and experimented with protecting and fostering birds on their farms. Some reported great successes in reducing the prevalence of insects in their fields or rodents in their barns, and, perhaps wishing to distinguish themselves from FitzSimons’s depiction of the degenerating bird slayer, some attacked his detractors as selfish and antiscientific.^77^ Many zoologists aligned with the broader wildlife protection movement were likewise enthusiastic about his campaign. Zoologist Ernest Warren of the Natal [Other P-480] Museum in Pietermaritzburg expressed his delight to have found an ally in the cause of bird protection.^78^ Likewise, in the first half of the 1920s, Captain Guy Shortridge of the King Williams Town Museum (now Amat-hole Museum) expressed his support and erected a display in his museum detailing the economic utility of carnivora in controlling rodents.^79^ Although FitzSimons had argued that Africans were insufficiently “mentally evolved to be amenable to reason and persuasion in this matter,”^80^ other bird protectionists extended his initiative toward Africans farming in reserves. For example, in March 1922, lectures on “native agriculture” were offered at the South African Native College, likely by missionary Bernard Huss, in which birds were declared “our faithful friends.”^81^ In his courses delivered at “Marianhill Native Training College,”^82^ Huss likewise sought to teach African students that the bird was “a great friend of man.”^83^ Huss also publicized a series of articles in the quadrilingual English, isiXhosa, isiZulu, and Sesotho periodical *Umteteli wa Bantu*, a newspaper that had an extensive circulation in the reserves.^84^ Titled “Principles and Methods of Agriculture,” this series emphasized the need for farmers to preserve the “balance of nature.”^85^

## Rodent Control and the Imbrication of Human and Carnivora Population Health

Despite the controversy it aroused, FitzSimons’s narrative of ecological decline producing outbreaks of deadly diseases was timely and provided [Other P-481] a compelling explanation for the sudden appearance of plague in the veld. The alleged decline of birds appeared to be concurrent with the perceived degeneration of the countryside into a rodent-infested den of disease, rather than one of mechanized modernity. Mitchell was clearly following FitzSimons’s controversy and in 1921 argued that the “almost complete destruction of jackals, lynxes and cats” was partly to blame for plague.^86^ Despite his critique of FitzSimons, in a 1922 letter to Mitchell, Roberts conceded that the plague problem was a result of “the disturbance of nature caused by the extermination of indigenous carnivora.”^87^ Mitchell agreed wholeheartedly, and in 1924 wrote to the Cape Provincial Secretary stating that “the whole question of the protection of bird life” should be “reviewed” in light of the “plague, locusts and other insect” problems.^88^ As evidence, he cited FitzSimons, Shortridge, and a letter he received from a farmer based in Westminster, Orange Free State, who claimed that his “trees and love of birds” were acting as a powerful defense against plague, since “all the chief plague areas are those where there is the least natural cover for birds of prey.”^89^

In early 1925, Mitchell began intensely to push the cause of protecting rodent-devouring predators. In a circular to all local authorities of the Union of South Africa, he wrote that plague had been able to colonize most rural areas because “the balance of nature . . . has been seriously upset.”^90^ This was a result of the “development of maize farming, the clearing of bush and cover, and the destruction of jackals, wild cats, mole-snakes, owls, and other natural enemies of rodents…”^91^ In his conception, plague within the veld was a symptom of a greater environmental illness and restoring balance to nature would bring the disease under control. For plague-carrying rodents to be excluded from spaces of settler agriculture, their predators had to be included on farms. In formulating a rural plague control strategy, Mitchell incorporated the “natural enemies” of rodents into his strategy as human allies. Not only hawks and owls, but also wildcats, and semiferal domestic cats, were deemed to be friends of the farmer, to be invited into settler spaces, and explicitly protected and [Other P-482] fostered.^92^ Once regarded as pests, Mitchell sought to transform these animals into agents of public health.

Humans, likewise, were integrated into Mitchell’s epidemiological strategy.^93^ Repopulating the veld with carnivora would take time, and farmers needed to compensate for their destructive actions by assuming the functions that rodent predators once had in maintaining a healthy balance of nature. Once again, African methods of pest control seemed to provide a model for white rural health. From circulars sent to magistrates across the country, Mitchell learned that some African areas, in contrast to white agricultural areas, were free of plague. The Magistrate of Tabankulu, for example, argued that in his “entirely Native district,” “Like every other wild animal with four legs, the rat lives a precarious existence. . . . The little herd boys are out all day with dogs, always on the watch for rats which they kill and devour. The rats are kept well down by their natural enemies such as cats, hawks, owls, etc., of which there are a considerable number.”^94^ Here, Africans were depicted as “natural enemies” of rats with prolific rodent hunting skills, who had also managed to preserve cats, hawks, and owls. White settlers, by contrast, supposedly lacked the natural knowledge of rodents needed to seek and destroy them, and viewed their predators as “vermin.”^95^ To account for this alleged ignorance, Mitchell and Roberts initiated an “experiment in propaganda” in 1925–28.^96^ This comprised a series of English and Afrikaans lectures delivered by Roberts and FitzSimons in “Plague areas” in the Transvaal, Free State, and Cape; the distribution of pamphlets on the natural history of veld rodents; and the exhibition of English, Afrikaans, isiXhosa, isiZulu, and Sesotho propaganda posters juxtaposing images of death with gerbilles, multimam-mate mice, and springhaases.^97^ Roberts and FitzSimons were tasked with explaining to farmers that their own destructive impulses were to blame for plague, impressing upon them the need to foster the “natural enemies” [Other P-483] of rodents, and teaching them to join such animals in killing rodents on their farms.^98^ Both zoologists explicitly told their audiences that they were to blame for the problem. For Roberts, farmers had created an overabun-dance of rodent food and shot out large “and often useful” animals while neglecting their duties in controlling rodents.^99^ While Roberts aimed to promote cohesion among white farmers through attempting to appeal to a shared sense of guilt, FitzSimons sought to pit farmers against one another. Here, he continued to rely on a stereotype of bird slayers as “ignorant, brutal, thoughtless or superstitious” in contrast to the enlightened farmer who protected birds on his lands.^100^

FitzSimons and Roberts’s lectures were complemented with a series of English and Afrikaans pamphlets on the natural history of veld rodents and methods of killing them, produced by William Powell. Such pamphlets were distributed to government rodent officers, who were tasked with identifying rodents, training farmers in rodent destruction, and distributing information on rodents to the public.^101^ In one, titled *Rodents: Description, Habits, and Methods of Destruction*, Powell argued that “jackals, all kinds of wild cats, owls, hawks, and mole snakes” had been “indiscriminately killed off” for years. This combined with the clearing of “bush and cover” in which such animals resided had “upset” the balance of nature.^102^ In addressing this, Powell argued that with exception of the despised jackal, these animals should be “protected and encouraged” on farms as far as possible.^103^ Unlike FitzSimons, Powell was in favor of a multispecies solution that combined poison, traps, rat proofing, fumigation, domestic cats, and wild birds to control rodents.^104^ Powell’s writings suggest that while birds and small carnivora would ordinarily maintain nature’s balance, the extraordinary situation produced by the modernization of agriculture meant that humans had to take on animal roles in the balance of nature and perform the functions owls, hawks, wildcats, and jackals once had. [Other P-484]

## Wildlife Protection as Public Health: Reception in Person, Periodicals, and Parliament

From the traces that remain of farmers’ perspectives on wildlife protection as a form of public health, there is little evidence to suggest that the propaganda campaign was a success. In certain towns, Roberts noted that some farmers had already been converted to the cause of bird protection, and were fostering rat-devouring wild owls on their properties by providing nesting boxes. Others seemed completely uninterested in or apathetic to the idea.^105^ Newspapers across the regions similarly suggest mixed success. The *Albert Times*, a paper based in Burghersdorp, a rural town within the plague-infected area, enthusiastically declared the lecture tours a great success.^106^ On the other end of the scale, *The Friend*, a Bloemfontein-based newspaper, declared that the Bloemfontein Town Hall (also in a plague-infected area) had been met with a “disgracefully poor audience” of mainly the converted—nurses and bureaucrats.^107^ The plague problem was covered extensively in *Umteteli wa Bantu*. On November 29, 1924, one reporter urged “Natives” to “join up” in the “war on rodents.” Among improving sanitation, keeping cats and dogs, and poisoning rats, the author advised that Africans “refrain from destroying birds and animals which prey on rodents” as doing so would “destroy” the “balance of nature.”^108^ Approximately a decade later, the same newspaper continued to publicize this advice: “preserve and foster natural enemies of rodents, such as wild cats, polecats, owls, hawks, and mole-snakes.”^109^ However, the extent to which this periodical was read by a Black agricultural audience remains largely unknown.^110^ This said, the case of renowned Zulu agriculturalist Robert Mazibuko does suggest that some African farmers were convinced by the campaign, or had already been attempting to preserve the balance of nature. Mazibuko, who studied agriculture under Bernard Huss in 1928, favored a holistic approach to farming that emphasized that “in nature everything is linked up or interconnected.” However, such ideas were not simply imparted upon him by Huss, but also learned “from our ancestors.” In an interview conducted in 1994, Mazibuko stated, “In African culture, the people always respected the land, the trees, the plants, the animals, birds and insects. For example, there [Other P-485] were many different birds that noone [*sic*] was allowed to kill—like owls, *nkombose*, *ngete*, eagles, vultures, storks and tickbirds. These birds were too useful to kill. You could not kill a secretary-bird either. It was important because it killed snakes.”^111^ Thus, it is possible that many of the same birds FitzSimons, Mitchell, Roberts, Huss, and others were imploring farmers to protect had already been under the ward of Mazibuko’s forefathers.

In contrast to general newspapers, in farming periodicals produced primarily for white audiences such as *Farmer’s Weekly* or its Afrikaans equivalent *Landbou Weekblad*, wildlife protection as a means of plague and rodent control received almost no attention. From a review of every issue of these two periodicals over the years 1925 to 1930, in thousands of articles, a mere forty-two were concerned with rats, and only eleven on plague.^112^ Of these, only three came from farmers. All others were produced by those involved in the experiment in propaganda.^113^

Ultimately, this mixed reception among various readers, writers, and lecture attendees did not prevent bird protectionists from making significant gains in national, provincial, and divisional legislature and regulations across the Union. Since 1922, various provincial and divisional councils had been passing ordinances and bylaws preventing the killing of birds. To name a few examples, in 1922, the Transvaal Province added insect and rodent-eating bustards, formerly on the Game List (which could be shot with license), to its list of “General Utility birds,” which were “entirely protected.”^114^ In 1923, Malmesbury Divisional Council likewise criminalized the killing of forty-eight species of birds, including owls and eagles.^115^ A year later, the Cape Province placed numerous birds of prey under provincial protection, including virtually all species of owl, two species of kestrel, and the Horsbrug’s Falcon.^116^ In the same year, King [Other P-486] Williams Town (now Qonce) was declared a bird sanctuary, in which bird destruction (of any kind) was prohibited.^117^

The return of bubonic plague, a disease associated with filth and primitivity that appeared to threaten the entire Union drew attention to the need for broadly applied legislature. In 1925, the first attempt to pass Union-wide laws for the protection of birds was made. In February, R. W. Close, an MP for Rondebosch, introduced the “Birds Export Prohibition Act,” which would criminalize the exportation of any bird outside of South Africa unless permission was obtained from the Minister of Agriculture.^118^ At its second reading on March 6, 1925, Close made it clear that the protection of birds was not only a matter of national pride, patriotism, and scientific importance, but one of public health. There “are many birds that have been shot out of this country,” Close argued, “birds whose natural food is the rodent of the country, and it is the rodents, as we know, that spread the plague.”^119^ The bill, he argued, was urgent and supported by “Prof. FitzSimons of Port Elizabeth” among others.^120^

At this reading, the plague problem would already have been fresh on the attending politicians’ minds, having been discussed at length the previous day. In this discussion, H. B. Papenfus, a representative for Hospital, had argued that bird protection offered a remedy for plague. In a series of questions to D. F. Malan, the Minister of Public Health, Papenfus suggested that the Department of Public Health needed to impose “rigid protection” on birds of prey.^121^ The “predatory wild birds known as raptores [*sic*]—to which belong the owls and hawks—,” he argued, “are great destroyers of rats and mice, but they have no protection under the law.”^122^ Acknowledging that these birds enjoyed partial protection across the Cape, he demanded that this be extended to the Transvaal and other provinces, and rigidly enforced, as this “balance of nature against rodents” was not something to be “interfered with.”^123^

Close’s bill was uncontroversial and achieved almost uniform support from all constituencies. Even Dr. A. J. Stals, the member for Hopetown, claimed that as “a representative of farmers I want to give it my full [Other P-487] support.”^124^ Jan Kemp, the Minister of Agriculture, likewise offered his blessing to the bill, claiming that he had “already asked” various provincial administrations to stop “the reckless killing of birds” on the grounds that “we kill far too many birds and do not appreciate sufficiently their value and the good services they render in connection with the combating of diseases and otherwise.”^125^ Close’s bill subsequently passed without modification and came into force on May 2, 1925. By June 1925, wildlife protection as a strategy of public health gained further legitimacy. Likely a direct result of this discussion in parliament, along with FitzSimons, Roberts, and Mitchell’s concurrent propaganda campaign, “ordinances for the protection of natural enemies of rodents, especially birds of the owl and hawk families and wild cats” had been passed in all four provinces.^126^

Perhaps emboldened by the success of bird protection as a public health policy, a year later Close attempted to pass stronger legislation. On January 26, 1926, he read an extraordinary bill that would criminalize bird destruction *of any kind* across the Union.^127^ The plague problem was Close’s primary justification for this bill. At its second reading on January 29, Close argued that bird protection was a matter of concern to every citizen of South Africa due to the fact that veld rodents were “spreading in the most extraordinary fashion the dire disease of plague.”^128^ In a speech that could easily have been taken from one of FitzSimons’s publications, Close stated that “one of the best means of exterminating these potential pests of the veld”—through the protection of wild birds—was “given to us by nature.”^129^ However, it had to be enforced evenly across the Union, since if a farmer in “some locality” delighted in “shooting off the eagles, hawks, owls, and other wild birds,” this might “create danger in other localities, which will come back to them, their households and friends, in the shape of disease-carrying rodents.”^130^ Criminalizing bird destruction, he argued, would “do more to keep the danger of plague at bay than any amount of money spent by Government to reduce the means of conveying the plague when too late.”^131^ [Other P-488]

This bill, however, unlike its predecessor, provoked bile and vitriol in its debate, exposing a diversity of views on what the “balance of nature” was, and how best it should be maintained. Close was attacked by politicians representing agricultural communities who insisted that birds were, with few exceptions, pests. I. P. van Heerden, the member for Graaff-Reinet, argued that Close, a Rondebosch man, had no place to be lecturing farming communities on this matter.^132^ H. Oost, the member for Pretoria North, agreed and claimed that the “large majority of wild birds in our country are a pest that ought to be exterminated,”^133^ and that legislation for useful birds already existed in the Transvaal and Free State since 1841, Natal since 1896, and the Cape since 1899.^134^

Other politicians were skeptical about a blanket ban and worried that this could actually exacerbate environmental and medical problems. For these men, human intervention in limiting and controlling animal populations was essential in the balance of nature, and humans had to keep raptor populations down or risk producing a plague worse than diseased rodents. P. W. van Niekerk of Waterberg, for example, argued that protecting hawks would be to the detriment of small birds, since for “every hawk that a farmer shoots” he will save “possibly, a thousand little birds.”^135^ Owls, on the other hand, were singularly useful in rodent control and in need of protection.^136^ For R. B. Waterston of Brakpan, protecting birds might simply deepen the looming environmental catastrophe: if protected in order to “drive out one pest” (rodents or insects), birds of prey might become worse pests than “the one driven out, as they have found in Australia with the sparrows…”^137^ Major G. R. Richards of Weenen, similarly, posed a eugenic argument against the bill. Although he agreed that “if you destroy birds you interfere with the balance of nature,” there were also “a number of birds which, if they are overprotected cease to be fertile and gradually die out.”^138^ Citing evidence from his own experience of farming, he noted that protecting partridges on one of his farms had actually caused their population to decline, unlike on his neighbor’s farm, where the birds were offered no protection. This, his response suggested, was [Other P-489] because it interfered with the struggle for life: it allowed “old birds” to remain and prevented the fertile and agile “young birds” from surviving.^139^

Others criticized and ridiculed the bill on the grounds that it would turn nearly every child in the country into a criminal (allegedly either bird killing or egg collection was universal among children) or would criminalize Christmas since the “turkey was once a wild bird.”^140^ Jan Kemp did not offer his blessing to the bill, unlike Close’s previous bill. In his view, the Union needed education rather than coercion. Children needed to learn “what birds are beneficial to the country,” and to love and protect these birds.^141^ Moreover, the bill was unnecessary: provincial administrations were empowered to pass bird protection laws and had already protected rodent and insect-killing birds in their localities. Kemp did not wish to appropriate their devolved powers.^142^

Close attempted to defend his bill by reading extensive passages from FitzSimons’s *Natural History of South Africa* and claiming that “there is in nature a balance, and we have to do our best to maintain that as far as we can” and that it would be a “very important step made to help, and, as far as possible maintain the balance of nature where we have had to do such things as the destruction of the jackal.”^143^ Sidestepping criticism, he argued that on grounds of the “sanitation, public health and interests of the community,” his bill needed to pass without modification.^144^

Close’s second bill hinged on the argument propagated by FitzSimons and his supporters that nature was fundamentally hospitable to white farmers, that virtually all birds maintained ecological stability, and that if the persecution of these animals stopped, they would bring balance to nature and plague would cease to be a problem. A blanket ban on killing these agricultural laborers and public health workers was therefore justified. Yet the parliamentary debate revealed that even though nobody was questioning the idea of a balance of nature, nor that certain bird species could serve as sources of free public health or agricultural labor, humans had to intervene in animal population dynamics to return balance to nature. For Close’s critics, the economies of nature and of agriculture were imbricated. While birds played a role in protecting human health and wealth, so too did humans need to safeguard the health and wealth [Other P-490] of nature. At times this meant killing predatory species.^145^ According to this conception of the balance of nature, the devolved powers of the provinces and divisional councils were actually critical in the quest of preventing plague and bringing balance. Local authorities knew their areas best and were already protecting those birds they deemed critical in controlling diseased rodents and insects. Further legislation was oppressive and unnecessary. As such, the bill failed to pass parliament, receiving thirty-five votes in favor, including from Deneys Reitz, the former Minister of Lands, and Jan Smuts, leader of the opposition. Fifty-eight voted against it, including D. F. Malan, the Minister of Public Health.^146^

Despite the failure of this bill, FitzSimons, Powell, and Mitchell continued to publicize propaganda, and further measures were taken to protect birdlife in numerous areas. The fact that local authorities were empowered to protect birds in their areas actually worked in their favor: by appealing directly to administrators and bureaucrats, they could circumvent parliament. In February, 1926, FitzSimons jubilantly announced that he had succeeded in prompting the Somerset East authorities to prohibit bird destruction in their area.^147^ Mitchell was delighted with this and expressed his support for bird protection as a measure of public health and his sorrow that “Mr Close’s Bill for the protection of birds has been thrown out by the House of Assembly.”^148^ By 1931, he succeeded in persuading the Port Elizabeth, and Walmer municipalities to declare themselves bird sanctuaries in which “all wild birds are protected under the law.”^149^ This seemed a national trend, particularly in towns and cities, and in a survey of local laws conducted in 1933, Roberts found that “the policy of protection birds in the urban areas is favoured in the great [Other P-491] majority of towns.”^150^ In a rapidly modernizing and politically changing country, a disease thought to be medieval had struck,^151^ and modernity itself seemed to be the root the problem. The unprecedented ability of humans to transform nature was rendering the veld a diseased landscape. In a decade of plague, as well as “Depression, drought and locust destruction,”^152^ an upset balance of nature seemed tangible, and restoring it became a means of promoting human health. In 1931, the *Rand Daily Mail* wrote that from “the economic point of view and from that of *public health*, the senseless and useless slaughter of most species of birds may be considered calamitous.”^153^

The evidence, however, suggests that bird protection laws were met with limited success. Both Close and Papenfus continuously pushed Kemp and Malan on matters of plague and bird protection and were horrified to learn that as of March 1927, permissions had been granted for as many as 20,781 birds to be exported, and not a single prosecution had been made under the Birds Export Prohibition Act.^154^ More problematically, the existing legislation did not actually forbid farmers from capturing protected birds on their farms and selling them. The result of this loophole was that thousands of birds were being caught with bird lime and traded on the market. Deneys Reitz, now Minister of Agriculture, thought that this “cruel” trade, peddled primarily by children trapping indiscriminately in the Transvaal Bushveld, constituted the “main evil” driving bird decline in South Africa, and introduced a “Wild Birds Protection Bill” into parliament in 1934 to criminalize it.^155^ Several politicians representing agricultural areas were opposed to the bill on the grounds that farmers relied upon trapping and selling or removing birds from their lands to protect their crops and livestock. They argued that wild birds also constituted a “plague.”^156^ On the other end of the scale, bird protectionists argued it did not go far enough, and insisted that to protect the balance of nature, [Other P-492] bird destruction of *any* kind needed to be prohibited as well.^157^ Some politicians, like FitzSimons, attempted to blame indigenous Africans for the problem, accusing them of roasting “thousands” of useful birds and eating them, and asserting the need for such legislature to be applied in Native Reserves.^158^ Despite criticism from both sides, Reitz’s bill passed parliament with few amendments, and came into force on April 13, 1934.^159^

Whether it was possible to enforce any of this legislation far into the rural interior of the country where plague continued to smolder is another matter. Nevertheless, the protection of carnivora in response to plague in the veld marks what is probably the first time a government public health institution officially framed wildlife protection as a strategy of disease control and a matter of human health. The terrifying prospect of bubonic plague creeping across the veld in bodies of gerbilles, multimammate mice, springhaases, and other rodents catalyzed a new understanding of public health in South Africa. The health of humankind needed to be viewed within a holistic network of human and animal population health, which could not easily be disentangled.^160^ Humans living in rural environments needed to find ways of coexisting with birds and carnivora to prevent the resurgence of the Black Death, and the collapse of nature’s harmonious balance.

## Conclusion

**Figure 2:**
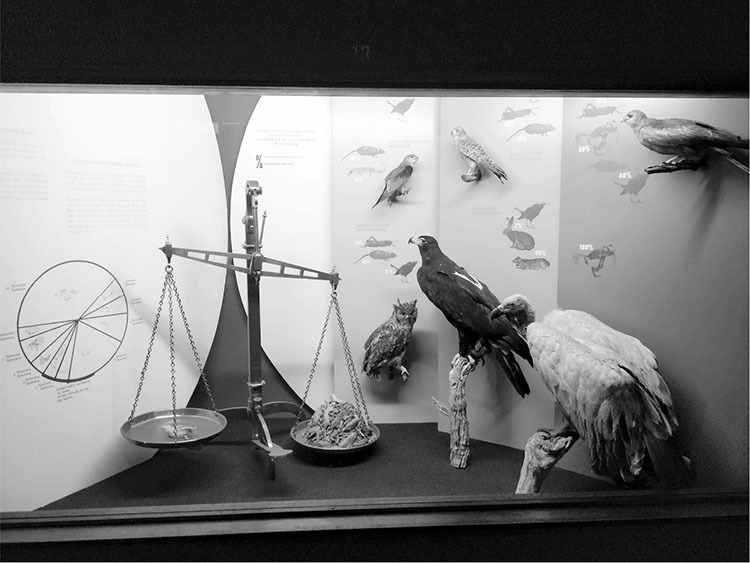
This display in the Austin Roberts Bird Hall is dedicated to birds “useful to man.” It argues that for each chicken killed, birds of prey kill an enormous number of rodents. The textual components forcefully argue that the net benefit of birds of prey outweighs their disadvantages to the farmer. It is a literal depiction of the balance of nature. Photograph taken by the author at the Ditsong National Museum of Natural History, Pretoria, in 2018.

As FitzSimons, Roberts, Mitchell, Close, and others proposed that wildlife protection was a means of preventing bubonic plague, they were partly articulating animal-protectionist views, and partly challenging a powerful state veterinary department that had argued the opposite. Their ideas shaped Mitchell’s policies, but less so his successors E. N. Thornton and E. H. Cluver. Thornton gradually turned away from Mitchell’s belief in an upset “balance of nature,” and in 1938, Cluver mobilized Charles [Other P-493] Elton’s animal population ecology as a strategy of plague management.^161^ Nevertheless, as late as 1941, officials in the public health department continued to advocate for the protection of birds of prey as a strategy of plague control and of obtaining free agricultural labor.^162^ To an extent such work continues to this day, as can be seen in the Ditsong National Museum of Natural History (Figure 2).

Between 1920 and 1938, five national parks were created in South Africa, and public opinion toward protecting wildlife continued to grow.^163^ This article suggests that concerns about health may have played a role in the South African conservation movement and proposes a new line of enquiry in the history of interwar African wildlife protection. As this case demonstrates, wildlife protection was not only a matter of preventing extinctions, capitalizing on “waste lands,” and creating zoological laboratories, but in some cases was key to the maintenance of the health of nature, and the associated health of humanity. African wildlife protection in the 1920s and 1930s was also a public health strategy.

The imbrication of animal and human population health in this case study demonstrates that the relationship between wildlife and white farmers in the 1920s and 1930s was not always one of separation, enclosure, and extermination. As conventional understandings of pests were challenged by FitzSimons, Mitchell, Close, and others, bird protectionists blurred the boundaries between human and animal, natural and cultural. For these scientists, in charting the balance of nature, whether in periodicals and propaganda or in the field itself, birds became agricultural laborers and public health workers. At times, humans took on former animal roles in the balance of nature. Species across the human-animal divide needed to build their worlds in synchronicity to preserve the health of the population of South Africa. [Other P-494]

Reflecting upon environmentalist thought in the 1920s and 1930s seems important in our current ecological moment. In advocating for the protection of birdlife, FitzSimons, Mitchell, Close, and others framed nature itself as fundamentally hospitable to white South Africa, but under assault by the same social forces that were seen to threaten the fabric of white minority rule: alleged white degeneration and the agency of indigenous Africans. In this way, there was a tacit acceptance that nature itself was providing services to sustain white health and agriculture, and that birds played a key role in delivering these services. Wildlife was framed in simplistic, anthropocentric terms as worthy of protection on account of the services it provided to whites, and those seeking to interfere with such services were criminals in need of fine or imprisonment. As such, the articulation of wildlife protection as a policy of public health relied on vilifying human underclasses and despised animals and blaming them for environmental and medical problems. [Other P-495]

This formula of framing wildlife protection as a service to the economy continues to inform certain sections of the African conservation movement to this day, whether through the discipline of ecosystem services identifying and calculating services rendered, or the creation of vast tourist spectacles of African fauna in game reserves. Many impoverished Africans continue to be demonized as “poachers,” and subsistence hunters trading bushmeat are sometimes blamed for outbreaks of zoonotic diseases.^164^ Selective outrage directs blame toward those against whom many already harbor prejudices, while protecting their own and the interests of wealthy factory farmers driving habitat destruction and risking outbreaks of further zoonotic diseases.^165^ Simultaneously, the demonization of insects, so fundamental to FitzSimons’s project may have contributed to the global collapse in insect populations. Ecologists are now acutely aware that many insects, thought to be largely harmful in the early to mid-twentieth century, “provide critical services within ecosystems” and their declining population might result in “a catastrophic collapse of nature’s ecosystems.”^166^ As we approach myriad contemporary medical and ecological crises, it is urgent that we resist measures that FitzSimons might approve of: simplistic propaganda campaigns demonizing particular species and peoples. Histories of environment, health, and politics can help us avoid these pitfalls. [Other P-496]

## Footnotes

1

FitzSimonsFrederick W The Natural History of South Africa: Birdsvol. 1, London Longmans, Green 1923 157.

2 Ibid., 104.

3 Ibid., 104.

4 See, for example, PageMichael LeHuge Global Extinction Risk for Insects Could Be Worse Than We ThoughtNew ScientistFebruary112019, https://www.newscientist.com/article/2193494-huge-global-extinction-risk-for-insects-could-be-worse-than-we-thought/; Sánchez-BayoFranciscoWyckhuysKris AGWorldwide Decline of the Entomofauna: A Review of Its DriversBiol. Conservation232April12019827

5
VidalJohnDestroyed Habitat Creates the Perfect Conditions for Coronavirus to EmergeScientific AmericanMarch182020, https://www.scientificamerican.com/article/destroyed-habitat-creates-the-perfect-conditions-for-coronavirus-to-emerge/; Tara Stoinski, “Opinion: How the Hard Lesson of Covid Could Help Gorillas,” CNN, July 31, 2020, https://www.cnn.com/2020/07/31/opinions/gorillas-conservation-covid-pandemic-stoinski/index.html; TollefsonJeffWhy Deforestation and Extinctions Make Pandemics More LikelyNature5847820August720201751763277014910.1038/d41586-020-02341-1

6 Some examples include HarrisonMarkClimates & Constitutions: Health, Race, Environment and British Imperialism in India, 1600–1850OxfordOxford University Press1999; NashLindaInescapable Ecologies: A History of Environment, Disease, and KnowledgeBerkeleyUniversity of California Press2006; SutterPaul S Nature’s Agents or Agents of Empire? Isis 98 4 2007 724 754.1831464310.1086/529265

7
MitmanGreggIn Search of Health: Landscape and Disease in American Environmental HistoryEnviron. Hist.1022005186; KohlerRobert EInside Science: Stories from the Field in Human and Animal ScienceChicagoUniversity of Chicago Press2019179180; WarrenJulianne Aldo Leopold’s Odyssey Washington, DC Island Press 2016 320 327 438.

8 Mitman, “In Search of Health” (n. 7), 186.

9
SellersChristopherTo Place or Not to Place: Toward an Environmental History of Modern MedicineBull. Hist. Med.92120183610.1353/bhm.2018.000029681547

10
RitvoHarrietCalling the Wild: Selection, Domestication, and SpeciesAfter Darwin: Animals, Emotions, and the MindRichardsonAngeliqueNew YorkRodopi2013268

11
CarruthersJaneThe Kruger National Park: A Social and Political HistoryPietermaritzburgUniversity of Natal Press1995; SittertLance vanHolding the Line: The Rural Enclosure Movement in the Cape Colony, c. 1865–1910J. Afr. Hist.431200295118; van SittertLanceRoutinising Genocide: The Politics and Practice of Vermin Extermination in the Cape Province c.1889–1994J. Contemp. Afr. Stud.341January22016111128; BeinartWilliamThe Rise of Conservation in South Africa: Settlers, Livestock, and the Environment 1770–1950OxfordOxford University Press2008; MutwiraRoben Southern Rhodesian Wildlife Policy (1890–1953): A Question of Condoning Game Slaughter? J. Southern Afr. Stud. 15 2 1989 250 262.

12 Carruthers, *Kruger National Park* (n. 11), 63–64; BrooksShirleyNational Parks for Natal? Zululand’s Game Reserves and the Shaping of Conservation Management Policy in Natal 1920s to 1940sJ. Natal Zulu Hist.22200473108; BrownKarenFrom Ubombo to Mkhuzi: Disease, Colonial Science, and the Control of Nagana (Livestock Trypanosomosis) in Zululand, South Africa, c. 1894–1953J. Hist. Med. & Allied Sci.6331July20082853221832688710.1093/jhmas/jrn003; CarruthersJaneInfluences on Wildlife Management and Conservation Biology in South Africa c.1900 to c.1940South Afr. Hist. J.5811January200777

13 Sellers, “To Place or Not to Place” (n. 9), 4.

14 The literature wildlife protection and zoology, hunting, tourism, and colonial power is vast. For zoological implications, see BontRaf DeA World Laboratory: Framing the Albert National ParkEnviron. Hist.222017404432. For hunting, see MacKenzieJohn The Empire of Nature: Hunting, Conservation and British Imperialism Manchester Manchester University Press 1988. For tourism, see Brooks, “National Parks for Natal?” (n. 12). For wildlife management as a facet of colonial power, see van Sittert, “Routinising Genocide” (n. 11); Carruthers, *Kruger National Park* (n. 11).

15
MitchellJ AlexanderPlague in South Africa: Perpetuation and Spread of Infection by Wild RodentsJ. Hygiene204192137810.1017/s0022172400034112PMC220705620474748

16 Ibid.

17 For literature on the Boer War, see GoochJohn The Boer War: Direction, Experience, and Image London Frank Cass 2000.

18
MitchellJ AlexanderBubonic Plague in Cape ColonySouth Afr. J. Sci.31January11905449

19

CarruthersJane National Park Science: A Century of Research in South Africa Cambridge Cambridge University Press 2017 57.

20

WordenNigel The Making of Modern South Africa: Conquest, Apartheid, Democracy London Wiley 2011 55.

21

DubeJohn L Presidential Address Ilanga Lase Natal February 23 1917.

22 Dawn Nell, “‘You Cannot Make the People Scientific by Act of Parliament’: Farmers, the State, and Livestock Enumeration in the North-Western Cape, c. 1850–1900” (M.A. thesis, University of Cape Town, 1998).

23

DubowSaul A Commonwealth of Knowledge: Science, Sensibility, and White South Africa Oxford Oxford University Press 2006 179.

24 Beinart, *Rise of Conservation in South Africa* (n. 11), 19.

25

BeinartWilliamDeliusPeter Introduction Putting a Plough to the Ground BeinartWilliamDeliusPeterTrapidoStanley Johannesburg Ravan Press 1986 1 55; Beinart, *Rise of Conservation in South Africa* (n. 11).

26 Beinart, *Rise of Conservation in South Africa* (n. 11), 21.

27 Beinart and Delius, “Introduction” (n. 25), 29; CarruthersJaneNattrassNicoliHistory of Predator-Stock Conflict in South AfricaLivestock Predation and Its Management in South Africa: A Scientific AssessmentKerleyGraham IHWilsonSharon LBalfourDavePort ElizabethCentre for African Conservation Ecology, Nelson Mandela University201830

28 Beinart, *Rise of Conservation in South Africa* (n. 11), 21.

29 For discussions of these arguments and the various legal transformations in agriculture in this period, see SittertLance van ‘Keeping the Enemy at Bay’: The Extermination of Wild Carnivora in the Cape Colony, 1889–1910 Environ. Hist. 3 3 1998 333 356; van Sittert, “Holding the Line” (n. 11); Beinart, *Rise of Conservation in South Africa* (n. 11); Carruthers and Nattrass, “History of Predator-Stock Conflict in South Africa” (n. 27).

30 Beinart, *Rise of Conservation in South Africa* (n. 11), 19.

31

WickinsPeter Agricultural Revolution in South Africa South Afr. J. Econ. Hist. 4 2 September 1 1989 109 130; van Sittert, “Holding the Line” (n. 11); Beinart, *Rise of Conservation in South Africa* (n. 11), 1–27.

32 Beinart, *Rise of Conservation in South Africa* (n. 11), 235–65.

33 Ibid., 366.

34

KeeganTimothy J Rural Transformations in Industrializing South Africa: The Southern Highveld to 1914 Johannesburg Ravan Press 1986 22.

35

DubowSaul Scientific Racism in Modern South Africa Cambridge Cambridge University Press 1995 16 140, 175.

36 For rodent-buffer zones, see Cape Town Archives Repository (KAB), 3/KWT 4/1/204. For elephant buffer-zones, see KAB, 1/UIT 17/18. For big-game and tsetse-fly buffer zones, see South African National Archives Repository, Public Records of Central Government since 1910 (SAB), LDE-N 4. See also MavhungaClapperton ChakanetsaThe Mobile Workshop: The Tsetse Fly and African Knowledge ProductionCambridge, Mass.MIT Press2018; van Sittert, “‘Keeping the Enemy at Bay’” (n. 29); van Sittert, “Routinising Genocide” (n. 11); Brown, “From Ubombo to Mkhuzi” (n. 12); BrownKaren Tropical Medicine and Animal Diseases: Onderstepoort and the Development of Veterinary Science in South Africa 1908–1950 J. Southern Afr. Stud. 31 3 September 2005 513 529.10.1080/03057070500202139PMC144092616628249

37

Van Der WattLize-Marie ‘To Kill the Locusts, But Not Destroy the Farmers’: Officials, Farmers and the Plagues of Pharaoh, c. 1920–1935 South Afr. Hist. J. 62 2 2010 356 383.

38

PhillipsHoward “Black October”: The Impact of the Spanish Influenza Epidemic of 1918 on South Africa, Archives Year Book for South African History, pt. 1 ( Pretoria Government Printer, South Africa 1990; PackardRandall M, review of “Black October”: The Impact of the Spanish Influenza Epidemic of 1918 on South Africa, by Howard Phillips J. Afr. Hist. 33 1 1992 159

39 Shirley Brooks, “Changing Nature: A Critical Historical Geography of the Umfolozi and Hluhluwe Game Reserves, Zululand, 1887 to 1947” (Ph.D. thesis, Kingston, Ontario, Queen’s University, 2001), 286–337.

40
PackardRandall MIndexing Immunity to Malaria in South Africa in the 1920s and 1930sAnthrop. Southern Afr.392May312016116130; PackardRandall M The Invention of the ‘Tropical Worker’: Medical Research and the Quest for Central African Labor on the South African Gold Mines, 1903–36 J. Afr. Hist. 34 2 1993 271 292.

41

LynterisChristos Ethnographic Plague: Configuring Disease on the Chinese-Russian Frontier London Palgrave Macmillan 2016 1.

42 While it would be interesting to know the extent to which these agricultural and political changes created fertile grounds for the spread of plague, such an exercise in historical ecology is beyond the scope of this paper, which favors examining the development of such ideas historically, rather than imposing modern science upon the past.

43 Austin Roberts to G. Breyer, May 1, 1920, Ditsong National Museum of Natural History, Austin Roberts Collection (ARC), 43.08.

44 Roberts to Breyer, May 1, 1920, ARC, 43.08; Roberts to Breyer, May 12, 1920, ARC, 43.08; Roberts to Breyer, May 19, 1920, ARC, 43.08; Roberts to Breyer, June 23, 1920, ARC, 43.08; Roberts to Breyer, September 13, 1920, ARC, 43.08.

45 Mitchell, “Plague in South Africa” (n. 15), 379–380.

46 “Plague Carriers of the Veld,” Lecture, Town Hall, Pretoria, February 3, 1925, ARC, 43.08.

47 J. Alexander Mitchell, “Report for the Year Ended 30th June, 1923” (Pretoria: Department of Public Health, 1923), 18–20; J. Alexander Mitchell, “Annual Report of the Department of Public Health Year Ended 30th June, 1924” (Pretoria: Department of Public Health, 1924), 27–34; J. Alexander Mitchell, “Annual Report of the Department of Public Health Year Ended 30th June, 1925” (Pretoria: Department of Public Health, 1925), 20–25.

48

PlugC Mitchell, Dr James Alexander (Public Health) S2A3 Biographical Database of Southern African Science April 23 2020, https://www.s2a3.org.za/bio/Biograph_final.php?serial=1936.

49 Mitchell, “Bubonic Plague in Cape Colony” (n. 18); SwansonMaynard W The Sanitation Syndrome: Bubonic Plague and Urban Native Policy in the Cape Colony, 1900–1909 J. Afr. Hist. 18 3 1977 387 410.1163221910.1017/s0021853700027328

50

PlugC FitzSimons, Mr Frederick William (Herpetology, Archaeology) S2A3 Biographical Database of Southern African Science February 21 2019, http://www.s2a3.org.za/bio/Biograph_final.php?serial=937.

51 Frederick W. FitzSimons, “Port Elizabeth Museum: Director’s Reports” (1905–39), Bayworld Museum Library (BML).

52 This is a distinct racial identity in South Africa, unlike its discriminatory counterpart in the United States, and the term remains in common, if at times contested use in present-day South Africa. It refers to mixed-race people with combinations of European, South Asian, Southeast Asian, Khoisan, or Nguni ancestry. For more information see AdhikariMohammad Burdened by Race: Coloured Identities in Southern Africa Cape Town UCT Press 2009.

53 Indigenous Africans were not explicitly invited to the museum, but there is no evidence to suggest that they were forbidden from visiting altogether.

54 See Uncatalogued Scrapbook, 1900s–30s, BML for numerous examples. FitzSimonsFrederick WThe House Fly: A Slayer of MenLondonLongmans, Green1915. FitzSimons maintained this public health connection into the 1920s. Museum Board Meeting Eastern Province Herald August 10 1922.

55 Davidson D. T. Jabavu, a Black professor at the South African Native College, for example, was a reader of the *Weekly*. See JabavuDavidson DTBlack Problem: Papers and Addresses on Various Native ProblemsLovedaleLovedale Press1920101. Afrikaans names and sentences in Afrikaans can be found regularly in the correspondence columns of this periodical. See, for example, van PletsenJ Sauer The Passing of the Locust Farmer’s Weekly January 12 1921.

56

FitzSimonsFrederick W Visitors Guide to the Typical Exhibits in the Port Elizabeth Museum Port Elizabeth Port Elizabeth Advertiser 1919.

57
FitzSimonsFrederick WThe Balance of NatureFarmer’s WeeklyAugust161922; FitzSimonsFrederick Birds and the Balance of Nature Farmer’s Weekly August 18 1920; FitzSimons, *Natural History of South Africa* (n. 1), 34–54, 100–107.

58 FitzSimons, *Natural History of South Africa* (n. 1), 2.

59 Ibid. See also Uncatalogued Scrapbook, BML, 1900s–30s. In making this argument, FitzSimons was drawing upon the works of ornithologists at the U.S. Bureau of Biological Survey, who since the 1890s had attempted to create a system for classifying the economic relations between birds and humans. Bird species, these ornithologists argued, could be classified as “Beneficial,” “Neutral,” or “Injurious,” based on whether they consumed noxious insects and “vermin,” or crops and livestock. He likewise appears to have been influenced by the work of Jan W. B. Gunning and Alwin K. Haagner at the Transvaal Museum in the 1900s, who argued that predatory birds played a key role in reducing miasmatic pollutants of the air. For a detailed analysis of this intellectual history, see Jules A. Skotnes Brown, “Pests, Knowledge and Boundaries in the Early Union of South Africa: Categorising, Controlling, Conserving” (Cambridge: University of Cambridge, 2020), 101–38.

60 FitzSimons, *Natural History of South Africa* (n. 1), 14, 37, 94; BucklandJamesThe Value of Birds to ManJ. Roy. Soc. Arts6332651915706

61 Unlike in the United States, and in the South African National Parks movement, where conservation and preservation approaches to wildlife management were distinct and often conflicting schools of thought, these categories were used interchangeably by FitzSimons. For example, on one page of his *Natural History of South Africa: Birds*, he insisted on the “preservation” of birds, while simultaneously advocating that farmers install “a very fine feeding shelter” to attract and foster birds—a strategy more commonly associated with conservation. My thanks to one of the reviewers for alerting me to this. FitzSimons, *Natural History of South Africa* (n. 1), 136.

62

FitzSimonsFrederick W Defeating Bird Raiders Farmer’s Weekly January 11 1922.

63

KrigeEileen J Agricultural Ceremonies and Practices of the Balobedu Bantu Studies 5 1 1931 207 239. Krige’s was the first systematic anthropological study of Balobedu agriculture, but similar technologies had already been suggested for use on government experimental farms in the 1900s. It thus seems likely that naturalists were learning from African farmers and not the other way around. See Charles B. Simpson to Director of Agriculture, November 7, 1906; F. B. Smith to Simpson, November 13, 1906, Public Records of former Transvaal Province and its predecessors as well as of magistrates and local authorities (TAB), TAD, 633.

64 Krige, “Agricultural Ceremonies and Practices of the Balobedu” (n. 63), 228.

65 Ibid., 228.

66 For a summary of his methods, see FitzSimons, “Defeating Bird Raiders” (n. 62).

67 Dubow, *Scientific Racism in Modern South Africa* (n. 35), 140, 175.

68 “Bird Life of South Africa, Mr. FitzSimons’ Plea for Preservation,” 1927, KAB, PAN 2/1.

69 FitzSimons, “Birds and the Balance of Nature” (n. 57), 3009.

70 FitzSimons, *Natural History of South Africa* (n. 1), 139.

71 FitzSimons, *Natural History of South Africa* (n. 1), 214–18.

72 Frederick W. FitzSimons, “Port Elizabeth Museum: Director’s Report” (Port Elizabeth: Port Elizabeth Museum, 1921); Frederick W. FitzSimons, “Port Elizabeth Museum: Director’s Report” (Port Elizabeth: Port Elizabeth Museum, 1923); Frederick W. FitzSimons, “Port Elizabeth Museum: Director’s Report” (Port Elizabeth: Port Elizabeth Museum, 1924).

73 Frederick W. FitzSimons, “Port Elizabeth Museum: Director’s Report” (Port Elizabeth: Port Elizabeth Museum, 1927).

74
EalesWLFitzSimonsFrederick WFruit-Eating Birds and Sociable CaterpillarsFarmer’s WeeklyJune301920; BillingtonRolandBirds as EnemiesFarmer’s WeeklyAugust181920; ChristianaBirds as a ScourgeFarmer’s WeeklySeptember221920; DavelFRBirds and CropsFarmer’s WeeklyDecember291920; SparrowGrain-Eating BirdsFarmer’s WeeklyOctober271920; WillFarmerCommon SenseFarmer’s WeeklyAugust251920; beginnerStruggling Birds and Shot Farmer’s Weekly November 3 1920.

75

SmithCharles Birds, Poets and Farmers Farmer’s Weekly November 3 1920 1301.

76 For numerous rebuttals in manuscript form and in correspondence, see ARC 43.22. For a published account of his views, which does not explicitly target FitzSimons, see RobertsAustin The Protection of Birds in South Africa South Afr. J. Natural Hist. 4 5 1924 317 333.

77 Whether pro or against FitzSimons, farmers frequently published under pseudonyms or first names, which makes their language and identity difficult to discern. For example, we know nothing about “Blue Jay,” “Sparrow,” “Farmer Will,” or “Struggling beginner,” beyond the fact that they could read and write in English, had access to a few copies of the periodical, and could afford or source funds for postage. This makes it impossible to assume the racial and linguistic identities of such writers. However, in some cases, we can assume that writers with surnames such as “van Pletsen” were Afrikaans, while “Kidson” was likely Anglophone. As the reader may gather from this, and footnote 74, support and criticism does not appear to be polarized along Anglo/Afrikaner lines. KidsonGCInterference with NatureFarmer’s WeeklyFebruary91921; JayBlueThe Altar of FashionFarmer’s WeeklyAugust111920; JMG The Sublime in Agriculture Farmer’s Weekly June 30 1920; PletsenvanPassing of the Locust” (n. 55); Van P, “The Sentinel Owl: Patrolling the Heavens at NightFarmer’s WeeklyJanuary131926; McIlwriathER Protection of Birds Farmer’s Weekly April 7 1926.

78 Warren also thought that insectivorous birds such as swallows were critical in controlling mosquitos, and thus malaria—a disease endemic to parts of Natal. Warren to FitzSimons, February 13, 1926, August 1925–February 1926, Ernest Warren Correspondence Cabinet, KwaZulu-Natal Museum Library.

79 Amathole Museum Library, Uncatalogued Scrapbook, 1921–34. See also the bird protection display on the upper floor of the Amathole Museum, Qonce, South Africa. This was constructed in the 1920s or 1930s and was still in place when I visited in 2018.

80 FitzSimons, *Natural History of South Africa* (n. 1), 140.

81
Father HussImvo ZabantsunduJanuary241922; Native Summer School: Lectures on Native AgricultureImvo ZabantsunduMarch71922; HussBernard A Text Book on Agriculture London Longmans, Green 1921.

82 Huss, *Text Book on Agriculture* (n. 81), vi.

83 Ibid., 86.

84 Erlank argues that this periodical circulated widely in native reserves, but also notes that the extent to which it “appealed to more ordinary workers, farm labourers, or peasants is unclear.” ErlankNatashaUmteteli Wa Bantu and the Constitution of Social Publics in the 1920s and 1930sSoc. Dynamics451201986

85

HussBernard Principles and Methods of Agriculture Umteteli wa Bantu October 19 1924.

86 Mitchell, “Plague in South Africa” (n. 15), 379.

87 Austin Roberts to J. Alexander Mitchell, January 5, 1922, ARC, 43.08.

88 J. Alexander Mitchell to Cape Provincial Secretary, June 30, 1924, KAB, PAN 2/1.

89 Ibid.

90 “Plague: Its Control, Eradication, and Prevention” (1925), appended to C. N. Millard, Circular No. 12 of 1925, “Plague,” March 16, 1925, KAB, 3/KWT, 4/1/204.

91 Ibid.

92 Ibid; Mitchell, “Annual Report of the Department of Public Health Year Ended 30th June, 1925”, 25 (n. 47).

93 *Pamphlet No. 306: Measures Against Plague* (1924), distributed on December 13, 1924, KAB, 3/KWT, 4/1/204.

94 Magistrate of Tabankulu to Secretary for Public Health, February 3, 1925, KAB, 1/TBU, 6/37.

95 Such thinking can be found elsewhere in the 1920s. In the case of locust swarms, Africans who ate the insects as a delicacy were considered a “natural enemy” of the locust. Van Der Watt, “‘To Kill the Locusts, But Not Destroy the Farmers’” (n. 37), 380.

96 “The plague problem. An experiment in propaganda,” unpublished report in ARC, 43.08.

97 See ARC, 43.08 in general and esp. “Reuter’s Press Notice: Plague.” For the propaganda posters, see KAB, 3/SMT, 4/1/40 and 3/KWT, 4/1/204.

98 “Plague Carriers of the Veld,” ARC, 43.08.

99 “Report on Lecture Tour on the Subject of Plague Rodents” (1925), ARC, 43.08.

100

FitzSimonsFrederick W The Black Death Somerset Budget en Pearston Advokaat March 11 1926.

101

PowellWilliam Rodents: Description, Habits, and Methods of Destruction Pretoria 1925 1, archived in KAB, 1/TGS, 4/18.

102 Ibid, 18–19.

103 Ibid, 19. For an analysis of the despised jackal, see BeinartWilliamThe Night of the Jackal: Sheep, Pastures and Predators in the CapePast & Present1581February11998172206

104 Powell, *Rodents: Description, Habits, and Methods of Destruction*, (n. 101), passim.

105 “Report on Lecture Tour on the Subject of Plague Rodents,” ARC, 43.08.

106

Plague Hygiene Albert Times December 3 1925.

107

Strenuous Fight against Plague The Friend February 18 1925 7.

108

Plague Umteteli wa Bantu November 29 1924.

109

Rats Mean Death Umteteli wa Bantu March 28 1936.

110 Erlank, “Umteteli Wa Bantu” (n. 84), 86.

111

BlochJoanneMazibukoRobert The Tree Man: Robert Mazibuko’s Story Pietermaritzburg New Readers Publishers 1996, https://live.fundza.mobi/home/library/non-fiction-general-articles/the-tree-man-robert-mazibukos-story/.

112 These periodicals are not digitized. My strategy for identifying articles was to examine the contents pages for anything mentioning rats, plague, mice, and rodents and to read these articles. I may have missed some articles, and thus these figures should be taken as approximate.

113 *Farmer’s Weekly*, 1925–30; *Landbouweekblad*, 1925–30.

114
Proclamation: Game Animals and Game Birds Protected in Various Magisterial Districts of the Province of Transvaal during Open Season 1922Official Gazette of the Province of Transvaal38April191922192

115
Malmesbury Municipality: Protection of Wild BirdsProvince of the Cape of Good Hope Official Gazette854March301923416

116
ScheduleProvince of the Cape of Good Hope Official Gazette952December519241350

117 Acting Town Clerk, King Williams Town to FitzSimons, November 16, 1925, KAB, 3/KWT, 4/1/204.

118

Union of South Africa, House of Assembly Debates of the House of Assembly: Second Session, Fifth Parliament, 13th February to 15th April 1925, vol. 3, Cape Town Cape Times Limited 1925 21.

119 Ibid., 660.

120 Ibid., 659.

121 Ibid., 568.

122 Ibid., 567.

123 Ibid., 568.

124 Ibid., 663.

125 Ibid., 664.

126 Mitchell, “Annual Report of the Department of Public Health Year Ended 30th June, 1925” (n. 47), 25.

127

Union of South Africa, House of Assembly Debates of the House of Assembly: Third Session, Fifth Parliament, 22nd January to 8th June 1926, vol. 6, Cape Town Cape Times Limited 1926 27.

128 Ibid., 116.

129 Ibid., 117.

130 Ibid., 117.

131 Ibid., 117.

132 Ibid., 119.

133 Ibid., 121.

134 Ibid., 122.

135 Ibid., 126.

136 Ibid., 126.

137 Ibid., 127.

138 Ibid., 131.

139 Ibid., 131.

140 G. Reyburn in ibid., 129.

141 Ibid., 130.

142 Ibid., 129–30.

143 Ibid., 135.

144 Ibid., 136.

145 On one level, this could be read as a conflict between preservationist and conservationist approaches to wildlife management. Jane Carruthers has shown that such distinctions were important in the creation and management of Kruger National Park. However, such categories were never used by politicians participating in this debate. It is possible that in regard to wildlife protection *outside of game reserves*, many stakeholders were unconcerned with or even unaware of this distinction, however far more research is needed into this question. Jane Carruthers, *Kruger National Park* (n. 11), passim, esp. 5.

146 Union of South Africa, House of Assembly, *Debates of the House of Assembly* (n. 127), 6:136.

147 Frederick W. FitzSimons to J. Alexander Mitchell, February 6, 1926, SAB, GES 515, 408/11-413/11B.

148 J. Alexander Mitchell to Frederick W. FitzSimons, March 16, 1926, SAB, GES 515, 408/11-413/11B.

149 Frederick W. FitzSimons to Town Clerk, Uitenhage, January 16, 1931, KAB, 3/UIT, 4/1/4, F1.

150 Austin Roberts, “Preliminary Report Upon an Enquiry Regarding Bird Protection in Urban and Industrial Areas,” ARC, 43.23.

151 I am grateful to Guy Geltner for this observation.

152 Van Der Watt, “‘To Kill the Locusts, But Not Destroy the Farmers’” (n. 37), 356.

153

Bird Friends of the Farmer Rand Daily Mail September 17 1931, emphasis added.

154

Union of South Africa, House of Assembly Debates of the House of Assembly: Fourth Session, Fifth Parliament, 28 January to 29 Junevol. 8 Cape Town Cape Times Limited 1927 1833 1834. This said, Kemp acknowledged that “the greater number of these birds are of species to the export of which there is no objection” (1834).

155

Union of South Africa, House of Assembly Debates of the House of Assembly: Second Session, Seventh Parliament, 26th January to 28th Marchvol. 22, Cape Town Cape Times Limited 1934 136.

156 Ibid., 141–42.

157 Ibid., 142–44.

158 R. H. Stuben in ibid., 146; Rood in ibid., 143.

159

Wild Birds Protection Act Union Gazette Extraordinary 22 13 April 1934 ii iii. Reitz did not mention the plague problem in his speeches and responses to criticism. Nevertheless, this connection would already have been well known, and the passing of his bill would have complemented the interests of FitzSimons, Close, Powell, and Mitchell (had Mitchell lived past 1933).

160 Holism is a keyword in South African ecology and politics in this period and refers to Jan Smuts’s philosophical, political, evolutionary, and ecological concept of holism. See AnkerPeder Imperial Ecology: Environmental Order in the British Empire, 1895–1945 Cambridge, Mass. Harvard University Press 2001.

161 Edward N. Thornton, “Annual Report of the Department of Public Health Year Ended 30th June, 1932” (Pretoria: Government Printer, 1932); Edward N. Thornton, “Annual Report of the Department of Public Health Year Ended 30 June, 1933” (Pretoria: Government Printer, 1933); Edward N. Thornton, “Annual Report of the Department of Public Health Year Ended 30 June, 1934” (Pretoria: Government Printer, 1934); Edward N. Thornton, “Annual Report of the Department of Public Health Year Ended 30 June, 1934” (Pretoria: Government Printer, 1934); Edward N. Thornton, “Annual Report of the Department of Public Health Year Ended 30 June, 1936” (Pretoria: Government Printer, 1936); Edward N. Thornton, “Annual Report of the Department of Public Health Year Ended 30 June, 1937” (Pretoria: Government Printer, 1937); Eustace H. Cluver, “Annual Report of the Department of Public Health Year Ended 30th June, 1938” (Pretoria: Department of Public Health, 1938), 52.

162

DavisDavid HSThomasAD Gerbille Control: An Aid to Crop Protection and Plague Prevention Union of South Afr. Department of Agric. and Forestry Zoological Survey, Vermin Control Series, no. 1 1941 9

163 Carruthers, *National Park Science* (n. 19), 19–21.

164

GreenLesley Rock | Water | Life: Ecology and Humanities for a Decolonial South Africa Durham, N.C. Duke University Press 2020, xii, 9–11; Giles-VernickTamaraShould Wild Meat Markets Be Shut Down?SomatosphereMarch62020, http://somatosphere.net/forumpost/wild-meat-markets/; HogenboomMelissa Is Bushmeat Behind Ebola Outbreak? BBC News October 19 2014, sec. Health, https://www.bbc.com/news/health-29604204; Jani Actman, “Bushmeat: How Wild Meat Can Be a Threat to Wildlife and Human Health,” National Geographic, June 19, 2019, https://www.nationalgeographic.com/animals/reference/bushmeat-explained/.

165 For a critique of the “hypocritical at best, and possibly outright racist” discussions surrounding COVID-19 and conservation, see SantanaCarlosCOVID-19, Other Zoonotic Diseases and Wildlife ConservationHist. Philos. Life Sci.424October8202010.1007/s40656-020-00345-8PMC754257033030629

166 Sánchez-Bayo and Wyckhuys, “Worldwide Decline of the Entomofauna,” 17.

